# The effect of double W tension-reduced suture technique on the abdominal scars following the da Vinci robot-assisted gastrectomy for severely obese patients

**DOI:** 10.1186/s12893-023-01979-8

**Published:** 2023-05-09

**Authors:** Wanying Chen, Tao Jiang, Ziming Zhong, Xiaodong Wang, Yang Cao, Yujing Wu, Haiyang Gai, Lianbo Zhang, Yang Yu

**Affiliations:** 1 Department of Plastic Surgery, China-Japan Union Hospital of Jilin University, Changchun, Jilin Province 130000 China; 2 Hepatobiliary and pancreatic surgery, China-Japan Union Hospital of Jilin University, Changchun, Jilin Province 130000 China; 3 Department of Plastic Surgery, Jilin Academy of Traditional Chinese Medicine Sciences, Changchun, Jilin Province 130021 China

**Keywords:** Double W tension-reduced suture, Scar, da Vinci robot-assisted surgery, Ultrasound

## Abstract

**Objective:**

To analyze the effect of a new type of tension-reduced suture named “double W tension-reduced suture technique” on the abdominal scars following the da Vinci robot-assisted gastrectomy for severely obese patients.

**Methods:**

40 abdominal incisions following the da Vinci robot-assisted gastrectomy on severely obese patients from September 1st, 2021 to March 1st, 2022 were comprised in the study. 20 incisions were closed by the conventional full-thickness surgical suture as the control group, and 20 incisions were sewn up by double W tension-reduced suture as the double W group. The scars were assessed at the 1-month follow-up visit using the Vancouver scar scale (VSS), ultrasound and patient satisfaction. Meanwhile, digital photographs of scars were taken as well.

**Results:**

The VSS score was 6.80 ± 2.16 in the control group, while that of the double W group was 2.60 ± 1.89. The difference between groups was significant. Digital photographs showed that the scar color was not only light and close to the skin color, but also flat and soft in the double W group. Ultrasound showed that the fibers of subcutaneous tissue in the double W group were arranged neatly, the ultrasonic signal intensity was relatively uniform, and the tunnel was small without obvious lacunae. More patients were satisfied and very satisfied with scars in the double W group.

**Conclusion:**

Double W tension-reduced suture technique could significantly improve the appearance and reduce comorbidities of scars following the da Vinci robot-assisted gastrectomy for severely obese patients.

**Supplementary Information:**

The online version contains supplementary material available at 10.1186/s12893-023-01979-8.

## Introduction

Obesity becomes a public health problem. The worldwide prevalence of obesity has increased to the pandemic level [1]. Obesity is associated with dyslipidaemia, hypertension, diabetes mellitus, and cardiovascular diseases [2]. Surgical treatment of obesity is an effective therapy, especially for severe obesity [3], which induces the sustained loss of weight and reduction of comorbidities [4–7]. Robot-assisted surgery brings the benefits of minimally invasive surgery to the weight-loss surgery [8]. The da Vinci operating robot was approved by FDA in 2000, and currently is widely applied in bariatric surgery [9–12].

Scars are inevitable in the surgical procedures [13], causing poor cosmetic results and decrease in life quality [14]. Tension plays an important role in the wound healing, and the reduction of skin tension helps to prevent and mitigate the scars [15, 16]. For severely obese patients, the thick abdominal wall and excessive intra-abdominal fat produce a high skin tension, resulting in the increasing risk of scar formation [16]. In the present study, we developed a new type of tension-reduced suture, double W tension-reduced suture technique, to improve the appearance of the abdominal scars following the da Vinci robot-assisted gastrectomy for severely obese patients. We assessed the safety and efficacy of the novel suturing method using digital photographs, Vancouver scar scale (VSS), ultrasound and patient satisfaction, and the short-term follow-up visit demonstrated the encouraging effect.

## Methods

### Study design

The study included 40 abdominal incisions from severely obese patients who underwent the robotic gastrectomy assisted by da Vinci Si surgical system with five trocars (8 mm) from September 1st, 2021 to March 1st, 2022 in the Department of Weight Loss and Metabolism, China-Japan Union Hospital of Jilin University. The study was approved by the Ethics Committee of China-Japan Union Hospital of Jilin University. Informed consent was signed by all the patients. Double W tension-reduced suture and conventional full-thickness surgical suture were performed respectively to close the incisions. The digital photographs, Vancouver scar scale (VSS), ultrasound and patient satisfaction were utilized to evaluate the scars at the 1-month following up visit.

### Patients

Patients meeting the inclusion criteria were included in the study, (1) severely obese patients that volunteered for the da Vinci robot-assisted gastrectomy, (2) patients without liver and renal function disfunction, coagulation disorders, myasthenia gravis and other major illnesses, (3) patients who did not take aspirin and other vasoactive drugs in the week before the surgery, (4) patients without mental illnesses. The exclusion criteria were as follows: (1) patients that had cardiovascular and cerebrovascular diseases, (2) patients who cannot undergo minimally invasive surgery, (3) patients with gastrointestinal malignancy.

### Suture techniques

After the da Vinci robot-assisted gastrectomy, 40 abdominal incisions were obtained. Half were closed by the conventional full-thickness surgical suture as the control. Interrupted full-thickness skin and fat layer were sutured by 3 − 0 polyglycolic acid absorbable sutures. Dressing was changed for the incision every other day after surgery, and the stitches were taken out 2 weeks later. During this period, patients were told to pay attention to waterproofing and avoid strenuous exercise, and anti-inflammatory treatment was applied to prevent infection.

**Fig. 1:**
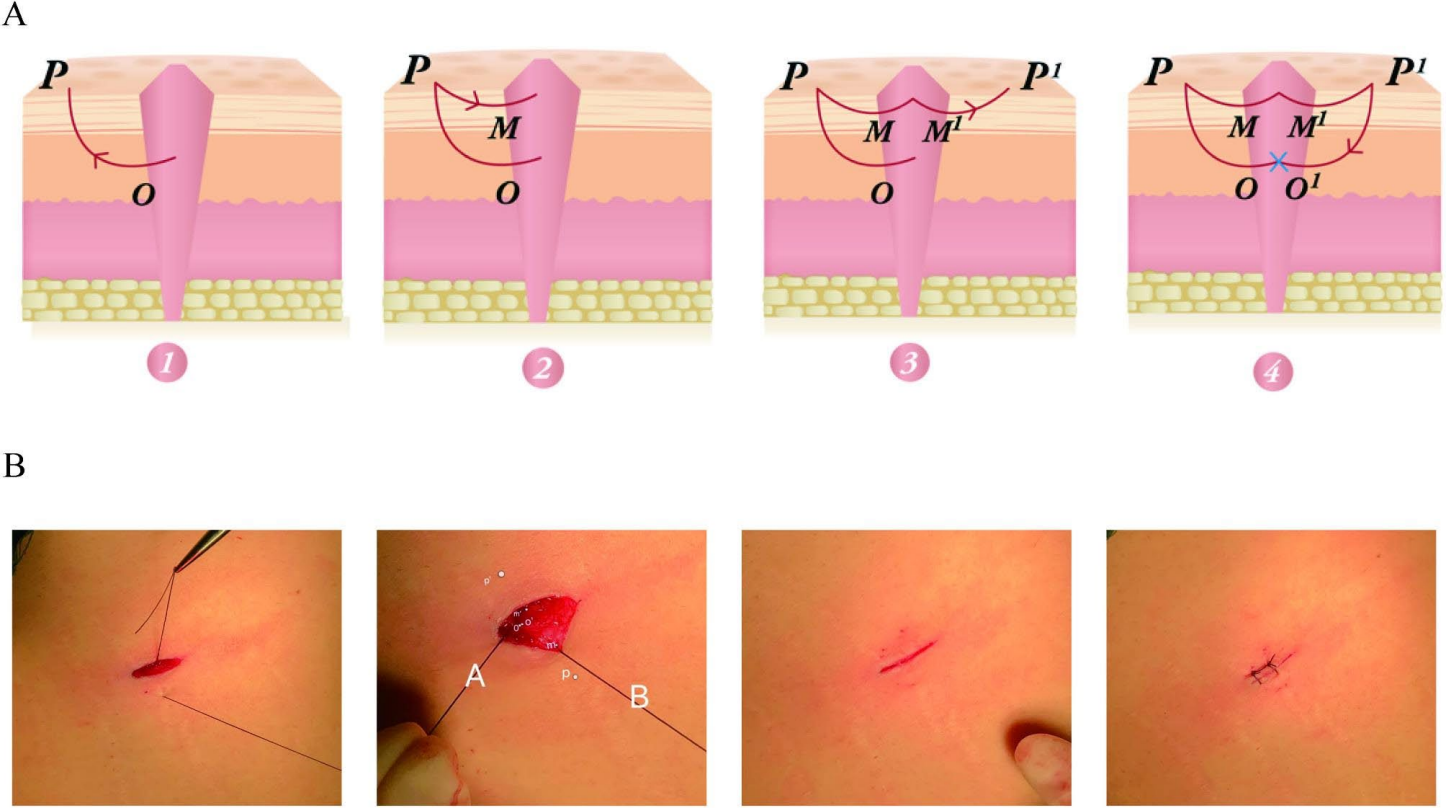
The procedure of double W tension-reduced suture technique. (A) Schematic. (B) Actual operation

The other half of the incisions were sewn up by double W tension-reduced suture. Figure 1 depicted the procedure. The incision was washed with normal saline and the surrounding skin was disinfected with iodophor, and then peritoneum and fascial layers were sutured in the customary manner. Sutured and trimmed the incision, and removed part of the 3 − 0 slow absorbable suture. Starting from the deep subcutaneous fat layer of the incision, the thread left in the deep layer was called line A, and the point in the deep layer was called point O. The needle took the direction of the suture (line B), and the position where line B penetrated the skin was called point P, which was about 1 cm from the incision. Point M in the middle of the dermis was located about 2 mm beneath the skin. The suture passed from point P through point M and back into the incision. Line A and line B were both located in the incision at that time. Subsequently, line B passed through the contralateral subcutaneous point M’ to the skin at point P’, and then passed through point O’ and back into the incision. Point O and point O’ were both located in the relative horizontal position of the deep layer of the incision. A surgical knot was tied in the deep subcutaneous tissue to avoid protruding from the skin surface to affect the scar. At that time, point P, P’, M, M’, O and O’ were located on a vertical plane, which was perpendicular to the tangent of the incision. Since the suture moved in an upper W shape and a lower W shape during the procedure, the new type of tension-reduced suture was named the double W tension-reduced suture technique. The postoperative operation was the same as that of the control group.

### Clinical assessment

At the 1-month follow-up visit, scars generated respectively by the conventional full-thickness surgical suture and double W tension-reduced suture were assessed by VSS score (from 0 to 15, the higher the score, the more severe the scar), ultrasound and patient satisfaction. Ultrasound was used to detect the subcutaneous tissue arrangement. Patient satisfaction was divided into four levels: unsatisfied, average, satisfied and very satisfied. Meanwhile, digital photographs of scars were taken as well.

### Statistical analysis

All the analysis was performed by SPSS (version 22.0). All data were reported as mean ± SD. The paired t-test was applied to analyze the differences between groups. P < 0.05 was considered as statistical significance.

## Results

**Fig. 2:**
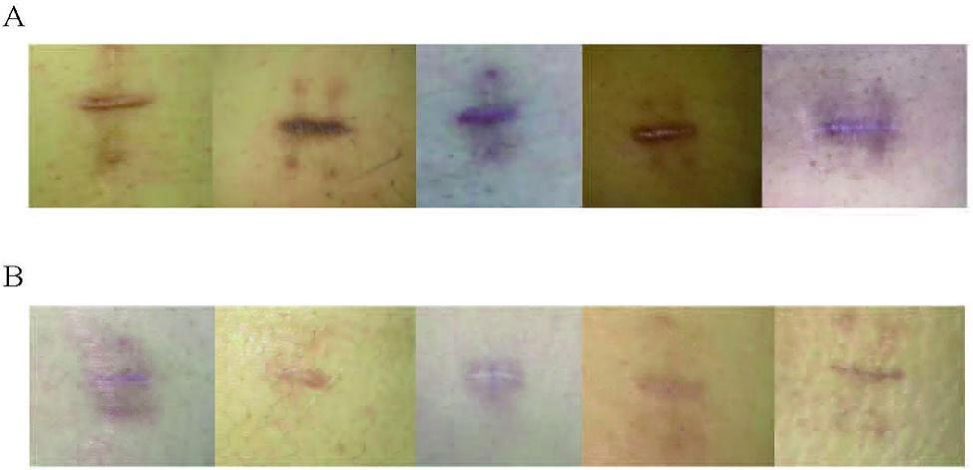
Digital photographs of scars in the control group (A) and the double W group (B)

**Fig. 3:**
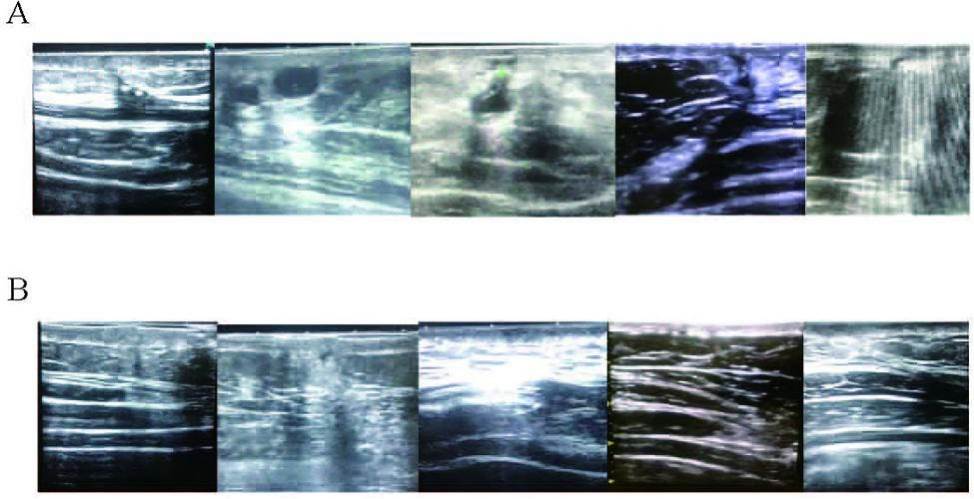
Ultrasound of scars in the control group (A) and the double W group (B)

At the 1-month follow-up visit, the VSS score was 6.80 ± 2.16 in the control group, while that of the double W group was 2.60 ± 1.89. The difference between groups was significant (p < 0.05). Figure 2 illustrated photographs of scars at the 1-month follow-up visit. The scars were not only light and close to the skin color, but also flat and soft in the double W group. In the control group, the scar was a bit too dark, and bulged obviously with hard tactile. Ultrasound showed that compared with the control group, the fibers of subcutaneous tissue in the double W group were arranged neatly, the ultrasonic signal intensity was relatively uniform, and the tunnel was small without obvious lacunae (Fig. 3). In terms of patient satisfaction, 11 patients (55%) were satisfied and 2 patients (10%) were very satisfied in the control group, while 10 patients (50%) were satisfied and 9 patients (45%) were very satisfied in the double W group. The detailed information for evaluation indices of scars was described in Supplementary Table S1-S4.

## Discussion

In the present research, we developed a new type of tension-reduced suture named double W tension-reduced suture technique. Compared with the conventional full-thickness surgical suture group, patients in the double W group had good shape of surgical scars, less pigmentation on the skin, less hypertrophic scar and less postoperative itch and pain, thus the double W group gained greatly better VSS score and higher patient satisfaction rate at 1 month after surgery. The double W tension-reduced suture had a large centripetal pulling force and a strong tension-reduction effect and long action time, which contributed to healing the incision and improving scar appearance and reducing comorbidities. Besides, it could reduce dead space to prevent hydrops from impeding the incision healing. Furthermore, the principle of the approach is so simple and realizable that it is easy to operate. Taken together, the double W tension-reduced suture technique could provide a kind of convenient, effective, and powerful tension-reduction effect for incisions during and after the operation, so that it could significantly improve the appearance and reduce comorbidities of the abdominal scars following the da Vinci robot-assisted gastrectomy on severely obese patients.

The selection of the appropriate suture material is important in the wound healing and scar formation. Compared with monofilament sutures, multifilament sutures have a higher coefficient of friction, strength and pliability, thus improving the knot security, however, they are more accessible to bacteria and fluid, leading to infection [17, 18]. Compared with natural suture materials, synthetic absorbable sutures reduce the undesirable tissue reactions to some extent [18]. Some studies reported that there was no significant difference in the cosmetic results and wound dehiscence between absorbable and non-absorbable sutures [19–21]. We used 3 − 0 polyglycolic acid absorbable multifilament suture to close the incisions. Polyglycolic acid has a high tensile strength, high knot security, and low melting temperature, making it a widely used suture material [22]. The tensile strength is reduced by 35% at 14 days and by 65% at 21 days. Polyglycolic acid has good biocompatibility, and undergoes hydrolysis that produces lower tissue reaction [23], and the degradation products have antibacterial effects [17].

We also utilized ultrasound as a tool for the postsurgical scar assessment in the current study and demonstrated its appropriateness. Ultrasound imaging showed that the scar tissue signal in the double W group was more uniform, and the incisions were more closely connected and closer to the normal tissue. While the control group had heterogeneous signal, and some incisions had obvious unhealed lacunae, which may further induce scar hyperplasia and cause discomfort to patients. As a dynamic and noninvasive diagnostic tool, ultrasound can objectively and accurately evaluate the features of postsurgical scars, which helps to plan the treatment and prevent postoperative complications. Therefore, the importance of ultrasound examination should be underscored, and ultrasound could be considered in the routine assessment and management of postsurgical scars [24].

There are several limitations in the study. The trial was small. Studies with a larger sample size and longer follow-up are needed to confirm the results. Some parameters of scars like width and length should have an accurate representation. Moreover, further research may focus on determining the feasibility of ultrasound in the postsurgical scars assessment.

## Conclusion

In conclusion, double W tension-reduced suture could significantly improve the appearance of the abdominal scars following the da Vinci robot-assisted gastrectomy on severely obese patients, and could effectively reduce comorbidities, suggesting that it has potential value of clinical application and spread.

## Electronic supplementary material

Below is the link to the electronic supplementary material.

Additional File 1: VSS score.
Additional File 2: Digital photographs of scars.
Additional File 3: Ultrasound of scars.
Additional File 4: Patient satisfaction of scars.

## Data Availability

The datasets used and/or analysed during the current study are available from the corresponding author on reasonable request.
