# Supplementary material for: The effect of double W tension-reduced suture technique on the abdominal scars following the da Vinci robot-assisted gastrectomy for severely obese patients
Source: BMC Surg. 2023 May 9;23:115. doi: 10.1186/s12893-023-01979-8 (PMC10170756; doi:10.1186/s12893-023-01979-8)
Supplement: Supplementary file 1 — Additional File 1: VSS score. Additional File 2: Digital photographs of scars. Additional File 3: Ultrasound of scars. Additional File 4: Patient satisfaction of scars. [file 12893_2023_1979_MOESM1_ESM.docx]

Table S1 VSS score.

| conventional full-thickness surgical suture | | | | | double W tension-reduced suture | | | | |
| --- | --- | --- | --- | --- | --- | --- | --- | --- | --- |
| Pigmentation | Height | Vascularity | Pliability | Total | Pigmentation | Height | Vascularity | Pliability | Total |
| 2 | 1 | 2 | 1 | 6 | 1 | 0 | 1 | 0 | 2 |
| 3 | 2 | 2 | 2 | 9 | 1 | 0 | 1 | 0 | 2 |
| 2 | 1 | 2 | 1 | 6 | 1 | 0 | 1 | 0 | 2 |
| 2 | 2 | 2 | 1 | 7 | 1 | 0 | 1 | 0 | 2 |
| 2 | 1 | 2 | 1 | 6 | 1 | 1 | 1 | 1 | 4 |
| 2 | 1 | 2 | 2 | 7 | 0 | 0 | 0 | 0 | 0 |
| 2 | 1 | 2 | 1 | 6 | 1 | 0 | 1 | 1 | 3 |
| 1 | 0 | 1 | 0 | 2 | 1 | 0 | 1 | 0 | 2 |
| 2 | 1 | 2 | 1 | 6 | 1 | 0 | 1 | 1 | 3 |
| 2 | 2 | 2 | 2 | 8 | 0 | 0 | 0 | 0 | 0 |
| 3 | 2 | 3 | 2 | 10 | 1 | 0 | 1 | 0 | 2 |
| 2 | 1 | 2 | 1 | 6 | 2 | 1 | 2 | 1 | 6 |
| 2 | 1 | 2 | 1 | 6 | 2 | 1 | 2 | 1 | 6 |
| 3 | 2 | 2 | 2 | 9 | 1 | 1 | 1 | 1 | 4 |
| 2 | 1 | 2 | 1 | 6 | 1 | 0 | 1 | 0 | 2 |
| 2 | 2 | 2 | 2 | 8 | 1 | 1 | 1 | 1 | 4 |
| 1 | 0 | 1 | 1 | 3 | 0 | 0 | 0 | 0 | 0 |
| 2 | 1 | 2 | 1 | 6 | 0 | 0 | 0 | 0 | 0 |
| 3 | 2 | 3 | 4 | 12 | 2 | 1 | 2 | 1 | 6 |
| 2 | 1 | 2 | 2 | 7 | 1 | 0 | 1 | 0 | 2 |

Pigmentation: from 0-3; Height: from 0 to 4; Vascularity: from 0 to 3; Pliability: from 0 to 5. The higher the score, the more severe the scar.

Table S2 Digital photographs of scars.

| conventional full-thickness surgical suture | | double W tension-reduced suture | |
| --- | --- | --- | --- |
| 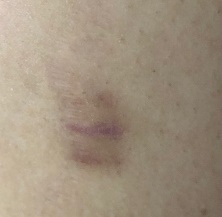 | 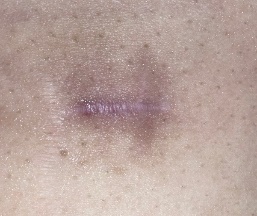 | 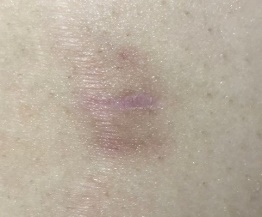 | 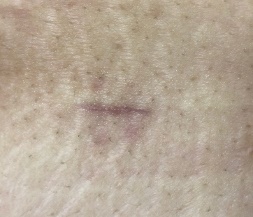 |
| 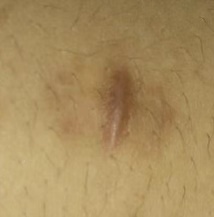 | 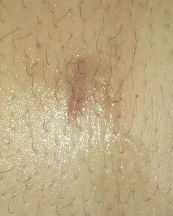 | 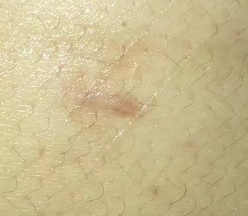 | 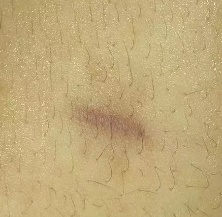 |
| 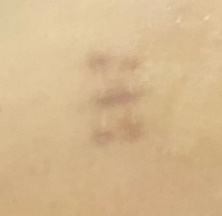 | 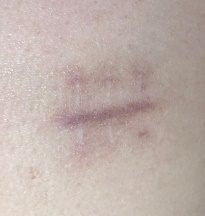 | 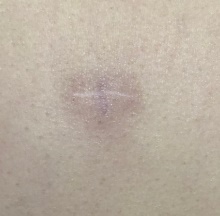 | 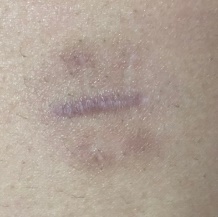 |
| 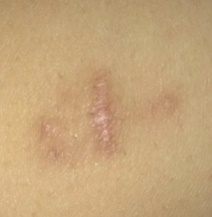 | 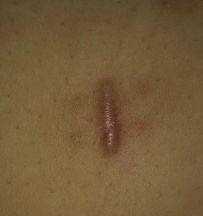 | 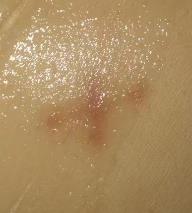 | 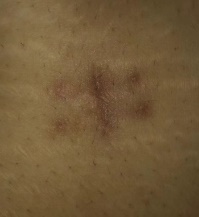 |
| 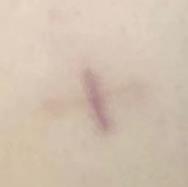 | 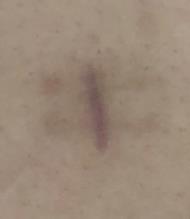 | 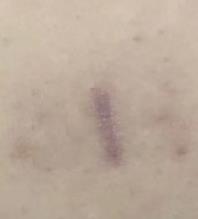 | 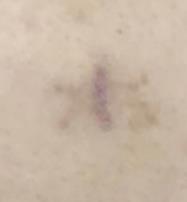 |
| 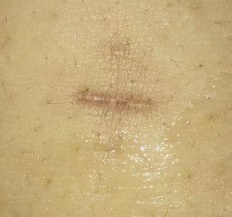 | 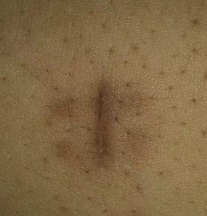 | 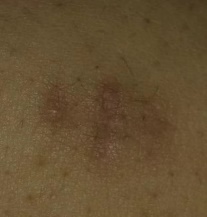 | 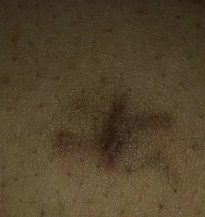 |
| 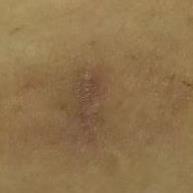 | 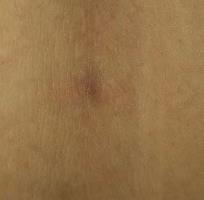 | 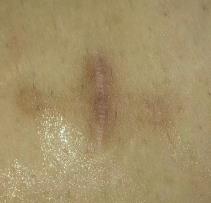 | 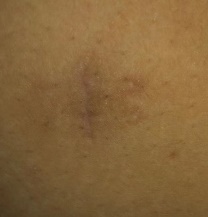 |
| 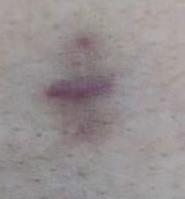 | 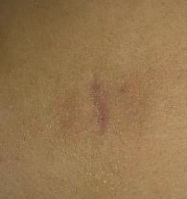 | 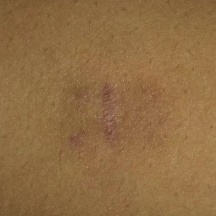 | 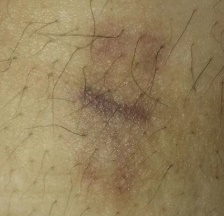 |
| 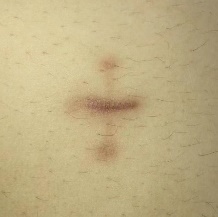 | 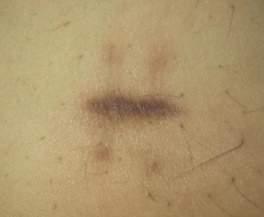 | 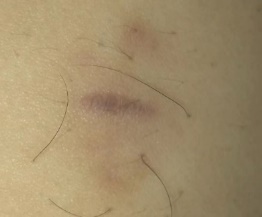 | 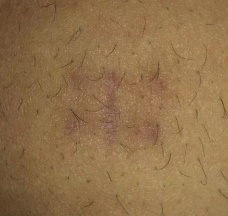 |
| 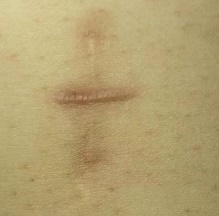 | 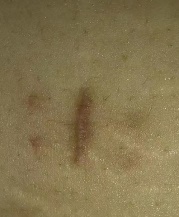 | 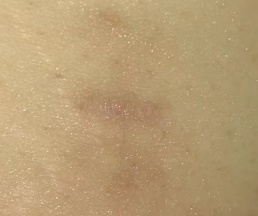 | 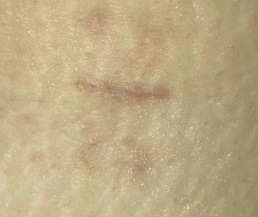 |

Table S3 Ultrasound of scars.

| conventional full-thickness surgical suture | | double W tension-reduced suture | |
| --- | --- | --- | --- |
| 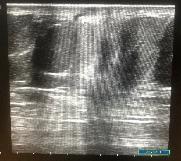 | 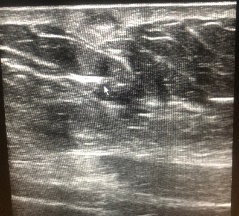 | 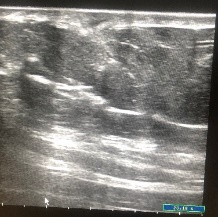 | 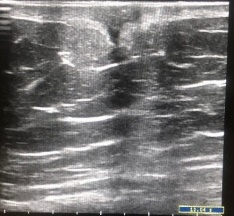 |
| 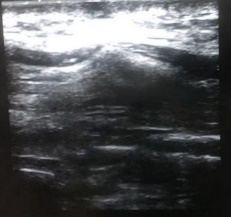 | 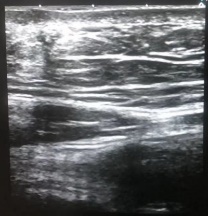 | 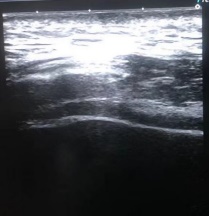 | 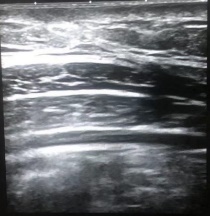 |
| 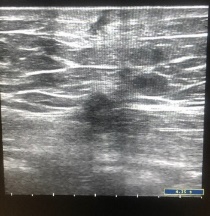 | 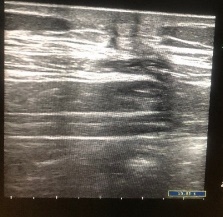 | 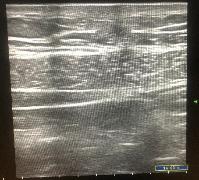 | 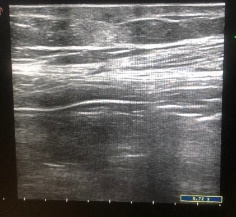 |
| 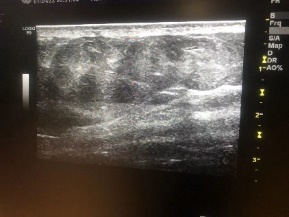 | 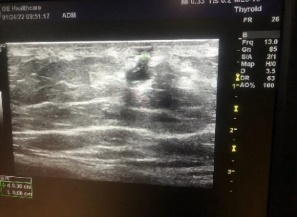 | 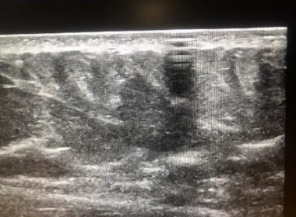 | 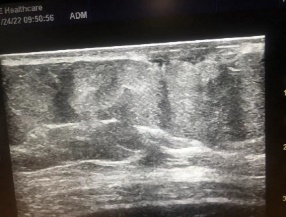 |
| 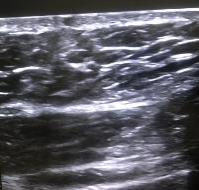 | 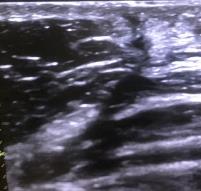 | 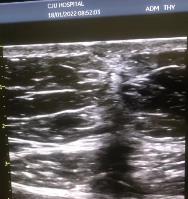 | 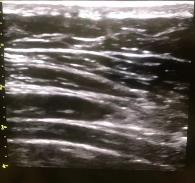 |
| 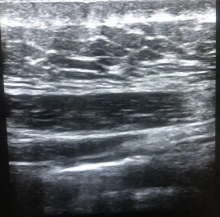 | 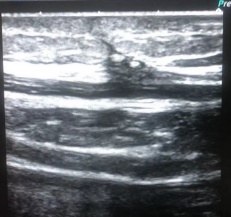 | 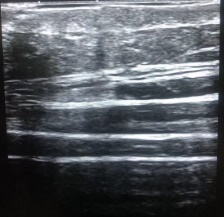 | 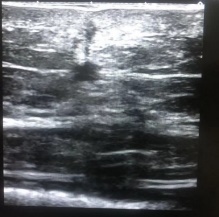 |
| 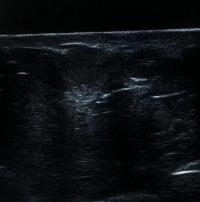 | 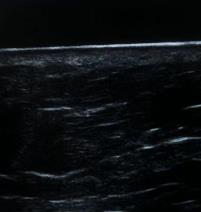 | 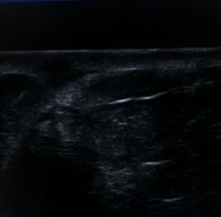 | 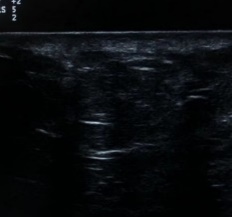 |
| 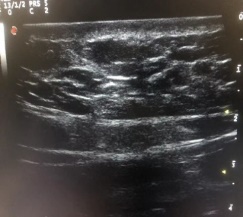 | 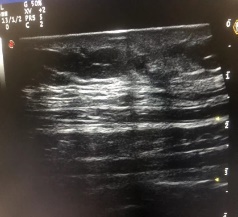 | 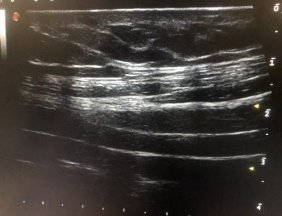 | 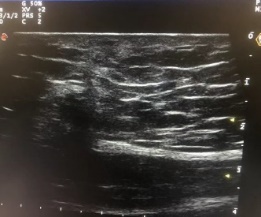 |
| 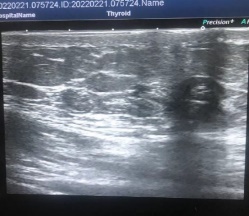 | 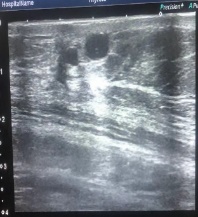 | 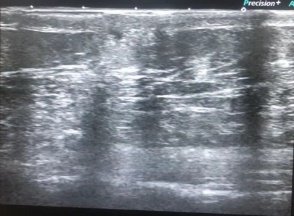 | 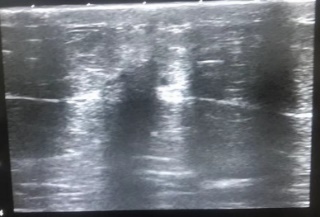 |
| 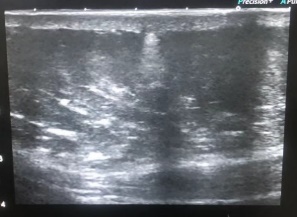 | 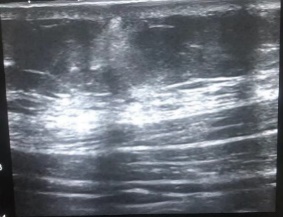 | 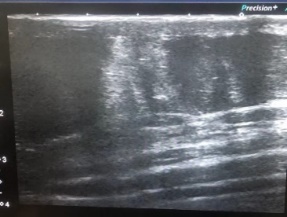 | 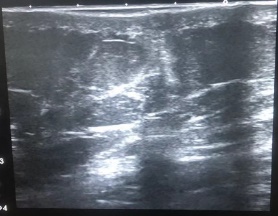 |

Table S4 Patient satisfaction of scars.

| conventional full-thickness surgical suture | | | | double W tension-reduced suture | | | |
| --- | --- | --- | --- | --- | --- | --- | --- |
| unsatisfied | average | satisfied | very satisfied | unsatisfied | average | satisfied | very satisfied |
| 3 | 4 | 11 | 2 | 0 | 1 | 10 | 9 |
